# Loss of Control Drinking In Alcoholics

**Published:** 1995

**Authors:** William R. Miller

**Affiliations:** William R. Miller, Ph.D., is the director of the research division of the Center on Alcoholism, Substance Abuse, and Addictions, and professor of psychology and psychiatry, University of New Mexico, Alburquerque, New Mexico

**Keywords:** self-control, AOD consumption, AOD dependence, expectancy, disease theory

**Figure:**
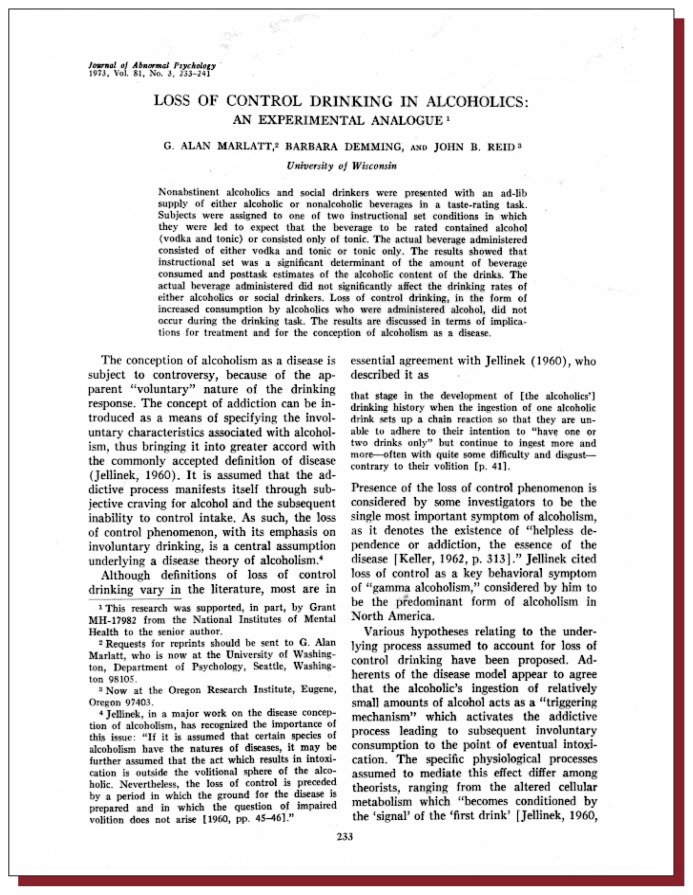
Marlatt, G.A.; Demming, B.; and Reid, J.B. Loss of control drinking in alcoholics: An experimental analogue. *Journal of Abnormal Psychology* 81(3):233–241, 1973.

When this landmark article was published in 1973, the dominant model of alcoholism considered it a dispositional disease, the cardinal symptom of which is inevitable loss of control whenever alcohol is consumed. The reasons for this loss of control over drinking were generally assumed to be physiological, an automatic and irreversible reaction to the chemical ethanol (pure alcohol). A few controversial voices in the alcohol field, including Jellinek (as expressed in his book *The Disease Concept of Alcoholism*, 1960), had questioned the scientific accuracy of the disease model as a universal description of alcoholism. Nevertheless, it was widely believed that the essence of alcoholism was a biomedical abnormality, inexorably rooted in the alcoholic’s physical constitution.

This innovative experiment by Marlatt and his colleagues put this assumption about alcoholism to the test by studying whether behavior changes resulted from the actual *presence* of alcohol or from the *belief* that alcohol was present. The authors introduced two novel research methods in this study, both of which inspired many subsequent studies. The first of these was the taste-rating task, in which subjects, made up of both alcoholics and social drinkers, were asked to taste and compare three ostensibly different beverages by rating them on a variety of descriptive adjectives such as “sweet” and “strong.” The actual purpose of the task was to study the amount and manner of drinking the subjects did without making them self-conscious that their drinking was being monitored. Later research has shown that this clever procedure does, in fact, mirror a person’s real-life drinking habits. It also is clear from two decades of subsequent studies that this unobtrusive measure is useful in gauging how a person’s drinking is affected by social and environmental factors.

The second innovative method introduced in this study was the balanced placebo design, which was made up of four groups of both alcoholics and social drinkers: Subjects in two groups were told that they were drinking alcohol, and subjects in two groups were informed that there was no alcohol in the beverage. Under these conditions, one-half of the subjects received alcohol and one-half did not. Marlatt devised an effective method to disguise the presence of alcohol so that the subjects could be convinced they were drinking alcohol when they were not (placebo group) or could be given alcohol without their being aware of it (balanced placebo group).

This study’s central finding was that the subject’s *belief* that he was drinking alcohol, rather than its actual presence, determined the amount he consumed on the taste-rating task. This effect was found for both alcoholics and social drinkers, although the difference was greater for alcoholics. As a result of Marlatt and colleagues’ demonstration, the balanced placebo design became a common research tool in the alcohol field. Dozens of subsequent studies have shown that it is the subject’s expectation that alcohol is present rather than the actual presence of alcohol that influences a broad range of social “effects” of drinking, including aggression, humor, sexual arousal, and anxiety. Such studies also have shown that it is mostly the amount, rather than the expectation, of alcohol that causes impairment on motor and memory tasks. In a direct replication of Marlatt and colleagues’ classic study, Stockwell and colleagues (1982) reproduced the findings in their study of dependent drinkers. However, Stockwell and colleagues reported that the presence of alcohol became a significant predictor of craving for severely dependent drinkers.

Besides these research innovations, publication of the study by Marlatt and colleagues also corresponded with what seems to have been a turning point for the involvement of psychologists and cognitive-behavioral models in the alcohol field. In the same year that this article was published, Gitlow expressed the then-dominant view that

the disease concept establishes alcoholism as firmly within the province of the medical profession, fixing responsibility for clinical care of the alcoholic and research into the nature of his suffering upon the physician and his paramedical partners (Gitlow 1973, p. 7).

Marlatt and colleagues’ classic study questioned, in both a direct and a symbolic way, the adequacy of this disease view of alcoholism. Perhaps the puzzling drinking behavior of alcoholics should be understood not as the inescapable product of a mysterious physical defect but rather as a modifiable behavior responsive to the same learning, cognitive, and psychosocial principles to which all behavior is responsive.

Research performed in the two decades after the publication of this landmark study amply demonstrated that drinking behavior is substantially influenced by many psychological factors. Elements such as therapist characteristics and client’s pretreatment and posttreatment psychosocial adjustment proved to be strong predictors of treatment outcome. The term “alcoholism” was omitted from formal diagnostic systems, such as the *International Classifications of Diseases* and the *Diagnostic and Statistical Manual of Mental Disorders*, in favor of the terms “alcohol abuse” and “alcohol dependence,” both of which came to be understood as continuous rather than dichotomous phenomena. Alcohol dependence itself was redefined as a syndrome with significant behavioral as well as physiological components (American Psychiatric Association 1987). Reviews of clinical outcome research found that psychological treatments headed the list of methods with the strongest scientific evidence of efficacy to treat alcohol-related problems.

Thus what was viewed in 1973 as an unitary medical disease is now understood in a broader and more complex context. This study opened one important door toward this integration of psychosocial and biomedical factors.
